# Editorials

**Published:** 1899-02

**Authors:** 


### EDITORIAL.

THE death of Dr. J. C. Meyer, Sr., of Cincinnati, Ohio, removed from the veterinary profession of our country one of the pioneers of our calling. A representative of our calling who, in the practice of his adopted vocation, left a strong impress of the value of his veterinary services over a wide territory; honored and respected in his own community for his valued services, he has won a higher reward from the profession in the greater lesson of appreciation he taught his people of its value to every community.

A VETERINARY staff, with a principal veterinarian having the rank of captain or major, should be the aim of the veterinary profession in the present proposed plan of reorganization of our army. This would insure safeguards to our army food-supply, and check such reckless expenditures and uncalled-for losses in purchasing army horses and provender for the same, which have become public scandal since the Spanish war.

### "NOW FOR THE SENATE."

THE House bill at Washington, known as the Hull bill, has successfully passed the lower branch of Congress and gone to the Senate for action. The efforts of the profession in having inserted there the rank of second lieutenant for those of our profession in the army was successful, and now it demands of us the most earnest and aggressive work in the Senate. Every veterinarian should now turn with renewed vigor his efforts to reach our Senators, and, in urging the retention of this clause, they should with equal force influence, so far as possible, the passage of the bill. There is danger of its failure in the short time remaining of the present session of Congress, and to go over to an extra session adds danger to our interests; while to wait for the meeting of Congress in December, or an extra session, makes our ultimate success more doubtful and leaves in jeopardy those who are to-day in tropical climates with their regiments, without protection or aught else that should be the safeguards of those who imperil their lives for their country's good. The work must be done now; it cannot wait, and your assistance may prove the balance that will win for our cause the recognition which we have worked and waited for so long.

### SCOTCH THESE BILLS IN NEW YORK.

SEVERAL bills have made their appearance in the legislative halls of the Empire State, all intended as enabling acts to persons unable to comply with the requirements of the present laws in force. Every one is destined to open the floodgates to let in a greater or lesser number of illiterate men who have posed as self-made veterinarians while earning their living as stable-bosses, superintendents, hostlers, and farriers. Not one of them is equipped for rendering intelligent professional service; and in the face of the State assisting in the education of men for the responsible duties of caring for the livestock interests, controlling the problems of a pure animal food-supply, restricting the dangers of those diseases communicable between man and the lower animals, there should be a quickened response by the veterinarians and their patrons to "scotch" such legislation beyond the hope of resurrection. There is a feeling developing that suggests the thought that these bills are brought forth for ulterior purposes, and they reflect seriously on the honor of those who father them in the respective legislative bodies.

---

WE ofttimes find among those whose veterinary education has been obtained through the lamp of experience, through the many vicissitudes that surrounded the obtaining of a veterinary education in America in years gone by, devout followers of their adopted vocation. Such was the life-history of the late Dr. J. C. Dustan, of Morristown, N. J. He loved his profession; he ardently supported its advancement; he loyally gave to association interests the best he could offer, and looked with the highest pleasure and gratification at its steady advancement. The loss of such men is always keenly felt, and their lives of usefulness should always teach an important lesson and charge the living with a stronger acceptation of the responsibilities that follow the entrance upon a professional life.

